# Multicomponent LBSap vaccine displays immunological and parasitological profiles similar to those of Leish-Tec® and Leishmune® vaccines against visceral leishmaniasis

**DOI:** 10.1186/s13071-016-1752-6

**Published:** 2016-08-30

**Authors:** Ludmila Zanandreis de Mendonça, Lucilene Aparecida Resende, Mariana Ferreira Lanna, Rodrigo Dian de Oliveira Aguiar-Soares, Bruno Mendes Roatt, Renata Alves de Oliveira e Castro, Maurício Azevedo Batista, Denise Silveira-Lemos, Juliana de Assis Silva Gomes, Ricardo Toshio Fujiwara, Simone Aparecida Rezende, Olindo Assis Martins-Filho, Rodrigo Corrêa-Oliveira, Walderez Ornelas Dutra, Alexandre Barbosa Reis, Rodolfo Cordeiro Giunchetti

**Affiliations:** 1 Laboratório de Biologia das Interações Celulares, Departamento de Morfologia, Universidade Federal de Minas Gerais, Belo Horizonte, Minas Gerais Brazil; 2 Laboratório de Imunopatologia, Núcleo de Pesquisas em Ciências Biológicas/NUPEB, Instituto de Ciências Exatas e Biológicas, Universidade Federal de Ouro Preto, Ouro Preto, Minas Gerais Brazil; 3 Laboratório de Pesquisas Clínicas, Programa de Pós-Graduação de Ciências Farmacêuticas, Universidade Federal de Ouro Preto, Ouro Preto, Minas Gerais Brazil; 4 Laboratório de Imunologia Celular e Molecular, Centro de Pesquisa René Rachou, Fundação Oswaldo Cruz, Belo Horizonte, Minas Gerais Brazil; 5 Laboratório de Biomarcadores de Diagnóstico e Monitoração, Centro de Pesquisa René Rachou, Fundação Oswaldo Cruz, Belo Horizonte, Minas Gerais Brazil; 6 Laboratório de Genômica de Parasitos, Belo Horizonte, Minas Gerais Brazil

**Keywords:** Visceral leishmaniasis, Vaccine, Leishmune®, Leish-Tec®, LBSap, Immunogenicity, Cytokines

## Abstract

**Background:**

In past years, many researchers have sought canine visceral leishmaniasis (CVL) prevention through the characterization of *Leishmania* antigens as vaccine candidates. Despite these efforts, there is still no efficient vaccine for CVL control.

**Methods:**

In the present study, we performed a pre-clinical vaccine trial using BALB/c mice to compare the effects of the multicomponent LBSap vaccine with those of Leish-Tec® and Leishmune®. Blood was collected to determine the frequency of peripheral blood cells and to evaluate hematologic and immunophenotypic parameters. Liver and spleen samples were collected for parasitological quantification, and spleen samples were used to access the cytokine profile.

**Results:**

When measuring total IgG and IgG1 anti-*Leishmania* levels after the third vaccination and *L. infantum* challenge, it was evident that all vaccines were able to induce humoral immune response. Regarding the innate immune response, increased levels of NK CD3^-^CD49^+^ cells were the hallmark of all vaccinated groups, whereas only the Leish-Tec® group displayed a high frequency of CD14^+^ monocytes after *L. infantum* challenge. Moreover, CD3^+^CD4^+^ T cells were the main circulating lymphocytes induced after *L. infantum* challenge with all evaluated vaccines. Importantly, after *L. infantum* challenge, splenocytes from the Leishmune® vaccine produced high levels of IL-2, whereas a prominent type 1 immune response was the hallmark of the LBSap vaccine, which presented high levels of IL-2, IL-6, TNF-α, and IFN-γ. The efficacy analysis using real-time polymerase chain reaction demonstrated a reduction in the parasitism in the spleen (Leishmune®: 64 %; LBSap: 42 %; and Leish-Tec®: 36 %) and liver (Leishmune®: 71 %; LBSap: 62 %; and Leish-Tec®: 48 %).

**Conclusions:**

The dataset led to the conclusion that the LBSap vaccination was able to induce immune and efficacy profiles comparable with those of commercial vaccines, thus demonstrating its potential as a promising vaccine candidate for visceral leishmaniasis control.

**Electronic supplementary material:**

The online version of this article (doi:10.1186/s13071-016-1752-6) contains supplementary material, which is available to authorized users.

## Background

Visceral leishmaniasis (VL), which is caused by *Leishmania infantum* (syn. *L. chagasi*) and is also known as Kala-azar, is a fatal infection if not treated successfully [1, 2]. Dogs play an important role in parasite maintenance because they are the main domestic reservoirs of *L. infantum* [3]. The best strategy to combat the spreading of disease would be the use of a vaccine to control canine visceral leishmaniasis (CVL). In recent years, several researchers have devoted their efforts to finding an efficient alternative for CVL prevention. However, no vaccine has proven to be effective [4–7].

In 2014, two commercially available vaccines were licensed by the Brazilian Ministry of Agriculture for use in dogs: Leish-Tec® (Hertape S.A., Juatuba, Brazil), which contains a recombinant amastigote stage–specific protein (rA2) of different *Leishmania* species plus saponin as an adjuvant [8–10], and Leishmune® (Zoetis, Campinas, Brazil), which is composed of semi-purified fucose-mannose ligand (FML) antigen glycoproteins from *Leishmania donovani* and saponin [11–13]. However, in November 2014, the Brazilian Ministry of Agriculture suspended the provisory license granted to the Leishmune® vaccine for failing to fully meet the requirements of a phase III vaccine clinical trial.

The choice of an appropriate experimental model is critical to the success of studies in leishmaniasis vaccinology. Several experimental models have been used in vaccine trials, including dogs, hamsters and mice [14, 15]. The murine models have several advantages such as: easy handling, low cost, short time of experimentation and wide availability of reagents for characterizing the immune response [15]. In this sense, the BALB/c mouse is a model highly used in preclinical studies anti-CVL.

Given the importance of the evaluation of innate and adaptive immune responses for understanding what response is associated with resistance and parasite control in VL-infected animals, this study aimed to compare the Leishmune® and Leish-Tec® vaccines with a patented vaccine candidate (LBSap) [16–18]. This study provides evidence that LBSap is a potential multicomponent vaccine for the prevention of VL because it induces parasite control and a protective immune response.

## Methods

### Animals, immunization protocol, and experimental challenge

Female BALB/c mice (6–8 week-old) received subcutaneous injections (100 μl/dose) of the vaccines at intervals of 14 days for a total of three injections. The animals were divided into four groups: Leish-Tec® (10 μg A2 and 50 μg saponin/dose); Leishmune® (150 μg FML and 50 μg saponin/dose); LBSap (60 μg *Leishmania braziliensis* antigen and 50 μg saponin/dose); obtained according [16]; and Control group, inoculated with 0.85 % sterile saline.

The LBSap vaccine was registered at the Industrial Property National Institute (Brazil) under patent number PI 0601225-6 (February 17, 2006). Leish-Tec® and Leishmune® were purchased and diluted according to each manufacturer at the time of immunization.

After 30 days of inoculum protocol, mice were challenged with 10^7^ promastigotes of *L. infantum* at the stationary phase in the lateral vein of the tail. Mice were euthanized 30 days after experimental challenge. The evaluations were performed at the following time points: before the first vaccination (BV); 15 days after the third saline [15^ASaline^] or vaccination (15^AVac^); and 30 days after experimental challenge (30^AChal^). Blood was collected to determine the frequency of peripheral blood cells and to evaluate hematologic parameters (BV, 15^AVac^, and 30^AChal^). Liver and spleen samples were collected for parasitological quantification, and spleen samples were used to access the cytokine profile (30^AChal^).

All experiments were performed using groups of five animals per evaluation time in two independent batches. The experiments showed similar results and the graphics are representative of one experimental batch (*n* = 5 per batch).

### Blood sample collection and differential leukocyte counts

The blood cell counts were determined using an electronic haematology particle counter (BC2800Vet, Mindray, Hamburg, Germany). Differential leukocyte counting was performed on Giemsa stained blood smears, and a total of 100 cells were counted.

### Humoral immune response

Antibody production was evaluated using a soluble lysate of *L. infantum* antigen (MHOM/BR/1972/BH46) (SLcA) and a conventional enzyme-linked immunosorbent assay. Briefly, 96-well microplates (MaxiSorp®; Nalge Nunc International, Rochester, NY) were coated with SLcA (at a concentration of 4.5 μg/ml) and after blocking with 2 % casein, serum samples were added at a dilution of 1:40 and the plates were incubated at room temperature. After a wash step, horseradish peroxidase (HRP)-conjugated goat anti-mouse IgG heavy and light chain (HRP-conjugated, anti-mouse, lot A90116P-29; Bethyl Laboratories, Montgomery, TX), anti-IgG1 (HRP-conjugated, anti-mouse, lot A90105P-31; Bethyl Laboratories, Montgomery, TX) or anti-IgG2a (HRP-conjugated, anti-mouse, lot A90107P-34; Bethyl Laboratories, Montgomery, TX) were added at dilutions of 1:3000, 1:2000, and 1:1000, respectively. The wells were then washed, substrate and chromogen (*o*-phenylenediamine; Sigma-Aldrich Co., St. Louis, MO) were added, and the absorbance was read at 492 nm on a Multiskan® MCC 340 (Labsystems, Helsinki, Finland) automatic microplate reader.

### Immunophenotyping of blood cells by flow cytometry

Immunophenotyping of blood cells was performed by flow cytometry. The cell markers used were monoclonal antibodies against CD14 (FITC anti-mouse CD14, clone Sa2-8/E00166-204; e-Bioscience, San Diego, CA), CD3 (PE-Cy5 anti-mouse CD3, clone 145-2C11/E060661630; e-Bioscience, San Diego, CA), CD4 (FITC anti-mouse CD4, clone GK1-5/E00078-133; e-Bioscience, San Diego, CA), CD8 (APC anti-mouse CD8a, clone 53.6-7/E070561330; e-Bioscience, San Diego, CA), CD19 (FITC anti-mouse CD19, clone NB19-1/E00184-1630; e-Bioscience, San Diego, CA), and CD49b (FITC anti-mouse CD49b, clone HMA2/E00340229; e-Bioscience, San Diego, CA). The antibodies were added to polystyrene tubes and 25 μl peripheral whole blood collected in EDTA was added to each tube. After homogenization in a vortex, the suspensions were incubated for 30 min at room temperature in the dark.

After erythrocytes lysis, the samples were centrifuged. The supernatant was discarded and the leucocytes were washed with phosphate-buffered saline. Afterward, the leukocytes were fixed with FACS FIX solution (10 g/l paraformaldehyde, 10.2 g/l sodium cacodylate, and 6.65 g/l sodium chloride, pH 7.2). Flow cytometry measurements were performed on a FACScalibur® instrument (Becton Dickinson, Mountain View, CA). The program CellQuest (Franklin Lakes, NJ) was used for data acquisition, and Flow Jo Software (Flow Cytometry Analysis Software 7.6.; Tree Star, Inc., Ashland, OR) was used for data analyses. The representative flow cytometry analysis strategy on Flow Jo Software can be seen in Additional file 1: Figure S1. Nonspecific binding was monitored by using fluorochrome-labeled isotypes to provide valid negative controls. Autofluorescence was monitored by the use of a negative control in which the cell suspension was incubated in the absence of fluorochrome-labeled monoclonal antibodies but in the presence of dilution and wash buffers.

### Cytometric bead array

The spleen cells were prepared as previously described elsewhere [19]. As specific stimulus we used the SLcA (25 μg/ml). After the experiment the supernatant was collected and stored in a freezer at -80 °C. The cytokine levels were measured by Cytometric Bead Array (BD Biosciences, San Jose, CA) according to the manufacturer’s recommendations. Concentrations of each test sample in picograms per milliliter (pg/ml) were calculated using FCAP software array v.1.0.2 (BD Biosciences, San Jose, CA).

The cytokines profile was demonstrated by the index of each cytokine obtained by dividing the values of the SLcA-stimulated culture and the unstimulated culture from the same animal. The index cytokine profile was also used to stratify the mice as low or high cytokine producers. For this analysis, the median of each cytokine was obtained; the animals with measurements that were above the median were considered high producers and the information was plotted on the radar chart.

### Real-time polymerase chain reaction

Real-time PCR was performed according to the protocol described in [17]. The pair of primers used (forward: 5ʹ-TGT CGC TTG CAG ACC AGA TG-3ʹ; reverse: 5ʹ-GCA TCG CAG GTG TGA GCA C-3ʹ) was described by [20] and targets the DNA polymerase gene of *L. infantum* (GenBank: AF009147), which is a single-copy gene, and amplifies a fragment of 90 bp. To verify the integrity of the samples, the same procedure was performed for the GAPDH gene (GenBank: AK168217.1). For amplification of the GAPDH gene, the primers used were forward 5ʹ-GAA ACC TGC CAA GTA TG-3ʹ and reverse 5ʹ-GGG AGT TGC TGT TGA AGT C-3ʹ. Reactions were processed and analyzed in an ABI Prism 7500-Sequence Detection System (Applied Biosystems, Walthan, MA, USA). The results were expressed as the number of amastigotes per milligram of spleen and liver.

### Statistical analysis

Statistical analyses were performed with GraphPad Prism 6 software (Prism Software, Irvine, CA). Normality of the data was demonstrated using a D'Agostino-Pearson normality test. One-way ANOVA followed by Tukey’s test was used to analyze the inter-groups (Control × Leish-Tec® × Leishmune® × LBSap) and intra-group differences (BV × 15^AVac^ × 30^AChal^). Inter-group differences were marked with letters corresponding to each experimental group, as follows: “C” as compared with the Control group; “LT” as compared with the Leish-Tec® group; “LM” as compared with the Leishmune® group and “LB” as compared with the LBSap group. Additionally, connecting lines are used to highlight intra-group differences at different time points. All differences were considered significant at *P ≤* 0.05.

## Results

### All vaccines induced increases in total IgG, IgG1 and IgG2a levels

**Fig. 1:**
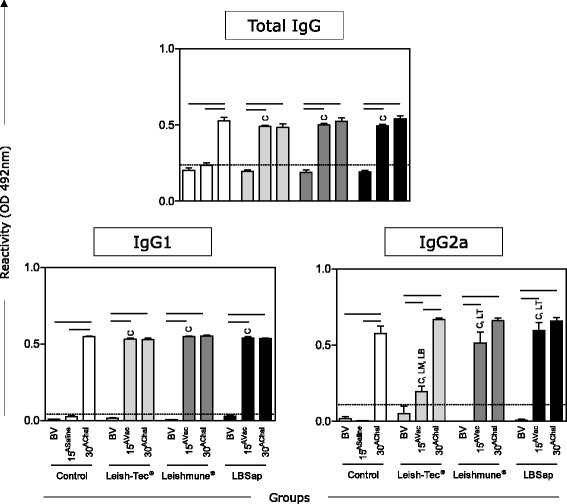
Comparative antigenicity by immunoglobulin analysis (total IgG, IgG1, and IgG2a) in serum. The x-axis displays the times at which the assays were conducted (before first vaccination [BV], 15 days after third saline [15^ASaline^] or vaccination [15^AVac^] and 30 days after experimental *L. infantum* challenge [30^AChal^]) using the different analyzed groups (*n* = 5 mice/group in two independent batches) (Control [□]; Leish-Tec® []; Leishmune® []; and LBSap [killed *L. braziliensis* vaccine plus saponin; ■]). The y-axis represents the mean enzyme-linked immunosorbent assay absorbance values determined at 492 nm in serum samples diluted 1:40. The cut-off edge is demonstrated by the dotted line for total IgG (0.24), IgG1 (0.04) and IgG2a (0.11). Connecting lines represent significant intra-group differences (*P* < 0.05) at the different time points. Inter-group differences at the same time point are marked by letters (C: Control group, LT: Leish-Tec® group, LM: Leishmune® group, LB: LBSap group)

Increases in total IgG, IgG1 and IgG2a were observed in all vaccinated groups at 15^Vac^ and 30^AChal^ as compared to BV (All statistical tests in this analysis had a *P* < 0.0001, except for Leish-Tec® group - IgG2a [BV *versus* 15^AVac^: *P* = 0.048]) (Fig. 1). It was also observed that IgG, IgG1 and IgG2a increased in all immunized groups at 15^Vac^ when compared to the Control group (All statistical tests in this analysis had a *P* < 0.0001, except in IgG2a Leish-Tec® (*P* = 0.002) *versus* Control group) (Fig. 1). Regarding the levels of IgG2a, there was an increase in Leishmune® (*P* = 0.0002) and LBSap (*P* = 0.003) groups at 15^Vac^ as compared with the Leish-Tec® group.

### Both Leish-Tec® and LBSap presented increased counts of lymphocytes after vaccination protocol

**Fig. 2:**
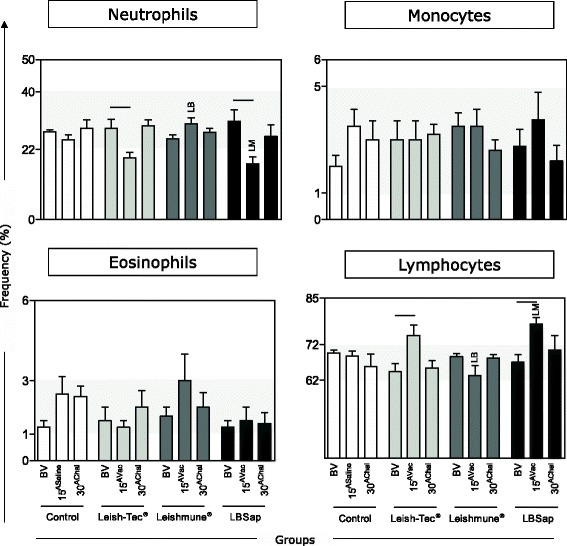
Differential leukocyte counts in peripheral blood leukocytes profile in BALB/c mice immunized with the multicomponent LBSap vaccine, Leish-Tec® or Leishmune®. The x-axis displays the times at which the assays were conducted (before first vaccination [BV], 15 days after third saline [15^ASaline^] or vaccination [15^AVac^] and 30 days after experimental *L. infantum* challenge [30^AChal^]) in the different analyzed groups (*n* = 5 mice/group in two independent batches) (Control [□]; Leish-Tec® []; Leishmune® []; and LBSap [killed *L. braziliensis* vaccine plus saponin; ■]). The y-axis represents the mean values (with standard deviations) of the frequencies of neutrophils, monocytes, eosinophils, and lymphocytes based on the hemogram. Connecting lines represent significant intra-group differences (*P* < 0.05) at the different time points. Inter-group differences at the same time point are marked by letters (LM: Leishmune® group and LB: LBSap group)

Differential leukocyte counts in peripheral blood leukocytes profile in BALB/c mice immunized with the multicomponent LBSap vaccine, Leish-Tec® or Leishmune® were carried out by conventional hematoscopy on Giemsa-stained blood smear. Significant decreases in total neutrophils were observed at 15^Vac^ in the Leish-Tec® (*P* = 0.050) and in the LBSap (*P* = 0.020) groups as compared to BV. LBSap group showed a decrease in total neutrophils at 15^Vac^ as compared with the Leishmune® (*P* = 0.004) (Fig. 2). Importantly, the total lymphocyte population displayed high counts at 15^Vac^ when Leish-Tec® (*P* = 0.048) and LBSap (*P* = 0.008) were analyzed as compared to BV (Fig. 2). At 15^Vac^ the LBSap group had an increase in total lymphocytes when compared with Leishmune® group (*P* = 0.004) (Fig. 2). Regarding the red series, no significant differences were observed (data not shown).

### Regarding innate immune response, increased levels of NK CD3^-^CD49^+^ cells were the hallmark of all vaccinated groups, whereas only the Leish-Tec® group displayed a high frequency of CD14^+^ monocytes after *L. infantum* challenge

**Fig. 3:**
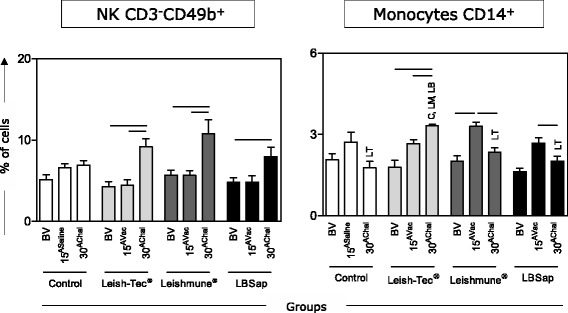
Immunophenotypic features of innate immunity (CD3^-^CD49b^+^ NK-cells and CD14^+^ monocytes) in BALB/c mice immunized with the multicomponent LBSap vaccine, Leish-Tec® or Leishmune®. The x-axis displays the times at which the assays were conducted (before first vaccination [BV], 15 days after third saline [15^ASaline^] or vaccination [15^AVac^] and 30 days after experimental *L. infantum* challenge [30^AChal^]) in the different analyzed groups (*n* = 5 mice/group in two independent batches) (Control [□]; Leish-Tec® []; Leishmune® []; and LBSap [killed *L. braziliensis* vaccine plus saponin; ■]). The y-axis represents the mean values (with standard deviations) of the frequency values in the lymphocytes or monocytes gate. Connecting lines represent significant intra-group differences (*P* < 0.05) at the different time points. Inter-group differences at the same time point are marked by letters (C: Control group, LT: Leish-Tec® group, LM: Leishmune® group, LB: LBSap group)

Immunophenotypic analysis was carried out by flow cytometry to characterize circulating innate immunity cells from BALB/c mice immunized with the multicomponent LBSap vaccine, Leish-Tec® or Leishmune®. An increase at 30^AChal^ for NK cells (CD3^-^CD49^+^) was observed in the Leish-Tec® (*P* = 0.015), Leishmune® (*P* = 0.030) and LBSap (*P* = 0.050) groups as compared to BV. Leish-Tec® (*P* = 0.005) and Leishmune® (*P* = 0.016) also showed that this increase at 30^AChal^ compared to 15^Vac^ (Fig. 3). The evaluation of circulating monocytes (CD14^+^) in the Leish-Tec® group showed increased counts at 30^AChal^ in comparison with both BV (*P* = 0.019) and 15^Vac^ (*P* = 0.003). The Leish-Tec® group also showed an increase at 30^AChal^ as compared to Leishmune® (*P* = 0.0002), LBSap (*P* = 0.0004) and control groups (*P* = 0.0002) (Fig. 3). Leishmune® presented high levels of CD14^+^ monocytes at 15^Vac^ as compared to BV (*P* = 0.001) and 30^AChal^ (*P* = 0.002). Similar results were observed in the LBSap group, which displayed increased counts of CD14^+^ monocytes at 15^Vac^ in comparison to 30^AChal^ (*P* = 0.034) (Fig. 3).

### CD3^+^CD4^+^ T cells were the main circulating lymphocytes induced after *L. infantum* challenge for all evaluated vaccines

**Fig. 4:**
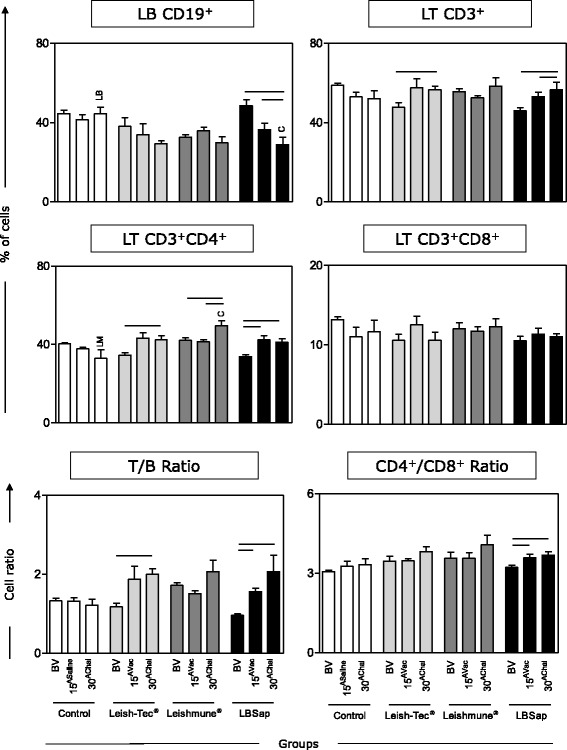
Immunophenotypic analysis of adaptive immunity (CD19^+^ B-cells; CD3^+^, CD3^+^CD4^+^ and CD3^+^CD8^+^ T-cells; T/B ratio and CD4^+^/CD8^+^ ratio in BALB/c mice immunized with the multicomponent LBSap vaccine, Leish-Tec® or Leishmune®. The x-axis displays the times at which the assays were conducted (before first vaccination [BV], 15 days after third saline [15^ASaline^] or vaccination [15^AVac]^] and 30 days after experimental *L. infantum* challenge [30^AChal^]) in the different analyzed groups (*n* = 5 mice/group in two independent batches) (Control [□]; Leish-Tec® []; Leishmune® []; and LBSap [killed *L. braziliensis* vaccine plus saponin; ■]). The y-axis represents the mean (with standard deviations) of the frequency values in the lymphocytes gate. Connecting lines represent significant intra-group differences (*P* < 0.05) at the different time points. Inter-group differences at the same time point are marked by letters (C: Control group, LM: Leishmune® group, LB: LBSap group)

Immunophenotypic analysis was carried out by flow cytometry to characterize circulating adaptive immunity cells from BALB/c mice immunized with the multicomponent LBSap vaccine, Leish-Tec® or Leishmune®. The Leish-Tec® and Leishmune® did not alter the frequency of CD19^+^ B-cells (Fig. 4). In contrast, LBSap vaccination led to a decrease in the frequency of B-cells at 15^Vac^ (*P* = 0.031) and 30^AChal^ (*P* = 0.005) as compared to BV. Moreover, a decrease of B-cells was observed at 30^AChal^ in the LBSap group as compared to the Control group (*P* = 0.014) (Fig. 4).

When evaluating total T-lymphocytes (CD3^+^), it was observed that the Leish-Tec® group presented an increase at 30^AChal^ in comparison with BV (*P* = 0.028) (Fig. 4). Furthermore, the LBSap group showed an increase in the frequency of T-lymphocytes at 15^Vac^ (*P* = 0.032) and 30^AChal^ (*P* = 0.044) as compared to BV (Fig. 4).

In the Leish-Tec® group there was an increase of T-helper lymphocyte (CD3^+^CD4^+^) at 30^AChal^ as compared to BV (*P* = 0.035) (Fig. 4). In the Leishmune® group, there was an increase at 30^AChal^ as compared to BV (*P* = 0.036) and 15^Vac^ (*P* = 0.044) (Fig. 4). Moreover, the Leishmune® group also presented an increase at 30^AChal^ as compared to the Control group (*P* = 0.016). Similarly, the LBSap group analysis showed sustained and increased counts of CD3^+^CD4^+^ T cells at both 15^Vac^ (*P* = 0.011) and 30^AChal^ (*P* = 0.009) as compared to BV (Fig. 4).

### LBSap displayed a higher T-lymphocyte/B-lymphocyte ratio in addition to an increased CD4^+^/CD8^+^ ratio after both vaccination and *L. infantum* challenge

The analysis of the CD3^+^ T cells/CD19^+^ B-cells ratio revealed an increase in the Leish-Tec® group at 30^AChal^ in comparison with BV (*P* = 0.002) (T/B ratio; Fig. 4). The LBSap group showed an increase at 15^Vac^ (*P* = 0.0002) and 30^AChal^ (*P* = 0.028) as compared to BV(Fig. 4).

Moreover, only the LBSap group showed an increase in the CD4^+^/CD8^+^ T-cell subset ratio at 15^Vac^ (*P* = 0.036) and 30^AChal^ (*P* = 0.014) as compared to BV (Fig. 4).

### After *L. infantum* challenge, splenocytes from the Leishmune® vaccine produced high levels of IL-2, but a prominent type 1 immune response was the hallmark of the LBSap vaccine, which presented high levels of IL-2, IL-6, TNF-α and IFN-γ

**Fig. 5:**
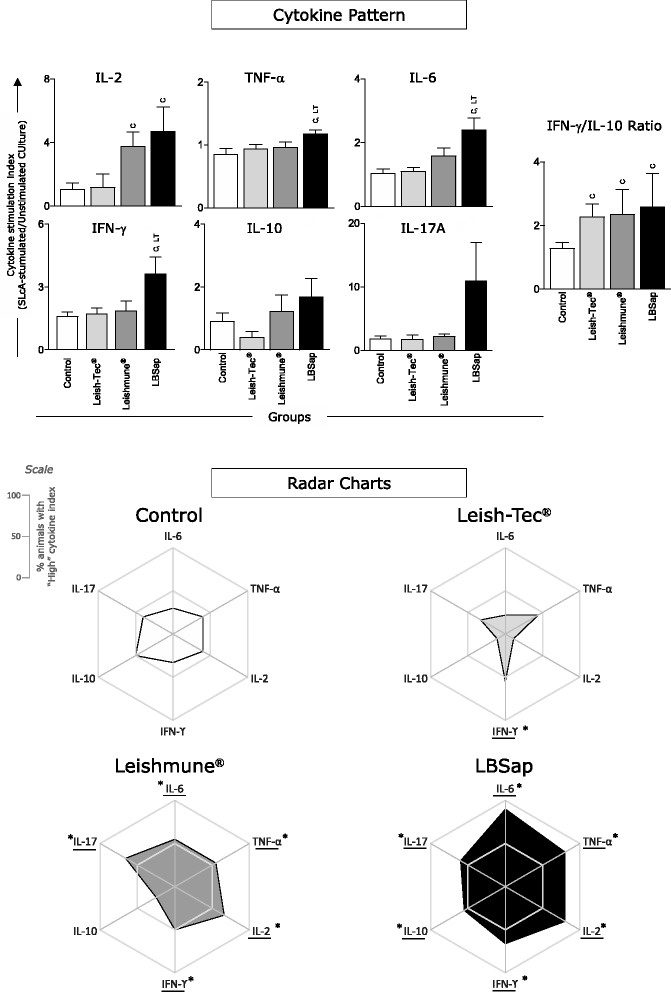
Overall proinflammatory and regulatory cytokine patterns secreted by splenocytes from BALB/c mice immunized with the multicomponent LBSap vaccine, Leish-Tec® or Leishmune®. In the upper panel, the x-axis displays the index (SLcA stimulated culture/unstimulated culture) of each analyzed cytokine (IL-6, TNF-α, IL-2, IFN-γ, IL-10, IL-17A) 30 days after experimental *L. infantum* challenge in the different analyzed groups (*n* = 5 mice/group in two independent batches) (Control [□]; Leish-Tec® []; Leishmune® []; and LBSap [killed *L. braziliensis* vaccine plus saponin; ■]). The y-axis represents the mean values (with standard deviations) of the stimulation index of analyzed cytokines. Significant inter-group differences are marked by letters (C: Control group and LT: Leish-Tec® group). The bottom panel displays radar charts of proinflammatory and regulatory cytokines from splenocyte supernatant culture. Each axis displays the proportion of high-producing cytokines in the following groups (*n* = 5 mice/group in two independent batches): Control (□); Leish-Tec® (); Leishmune® (); and LBSap (killed *L. braziliensis* vaccine plus saponin; ■)

Data analysis showed an increased IL-2 index in both Leishmune® (*P* = 0.011) and LBSap (*P* = 0.028) groups as compared to the Control group (Fig. 5, upper panel).

Furthermore, TNF-α analysis demonstrated high levels in the LBSap group as compared to the Leish-Tec® (*P* = 0.030) and Control (*P* = 0.011) groups (Fig. 5, upper panel). Similar results were observed with IL-6, which presented increased levels in the LBSap group when compared to the Leish-Tec® (*P* = 0.006) and Control (*P* = 0.003) groups (Fig. 5, upper panel).

We also observed an increase in the IFN-γ index in the LBSap as compared to the Leish-Tec® (*P* = 0.048) and Control (*P* = 0.026) groups (Fig. 5, upper panel). No significant differences were observed regarding IL-10 and IL-17A production (Fig. 5, upper panel). IL-4 production was below the limit of detection of the test (data not shown).

Additional analysis of pro-inflammatory/regulatory cytokine balance (IFN-γ/IL-10 index ratio) revealed a clear picture that all three vaccines [Leish-Tec®(*P* = 0.048), Leishmune® (*P* = 0.036) and LBSap (*P* = 0.050) as compared to the Control group] were able to trigger a cytokine balance shifted toward a pro-inflammatory pattern, with index ratio approximately twice higher for IFN-γ than IL-10 (Fig. 5, upper panel).

In addition, the frequency of the overall cytokine production was further evaluated by determining the proportion of high producers that were displayed on radar charts (Fig. 5, bottom panel). The Control group demonstrated low numbers of high producers of pro-inflammatory cytokines, and 50 % of animals presented as high producers of IL-10 (Fig. 5, bottom panel). Additionally, the Leish-Tec® group displayed a low frequency profile with high producers of pro-inflammatory and regulatory cytokines, although approximately 50 % of animals showed high IFN-γ production. Moreover, the Leishmune® group revealed a pro-inflammatory profile with approximately 50 % of animals being high producers of IL-6, TNF-α, IFN-γ, and IL-17, in addition to basal levels of IL-10. Furthermore, the hallmark of the LBSap group indicated a prominent type I immune response with a great frequency of animals (range, 60–90 %) displaying high production of IL-6, TNF-α, IFN-γ, and IL-17; approximately 50 % of animals showed high IL-10 production (Fig. 5, bottom panel).

### Leishmune® vaccination led to a reduction in parasite load in the spleen, whereas Leishmune® and LBSap presented low parasitism in the liver

**Fig. 6:**
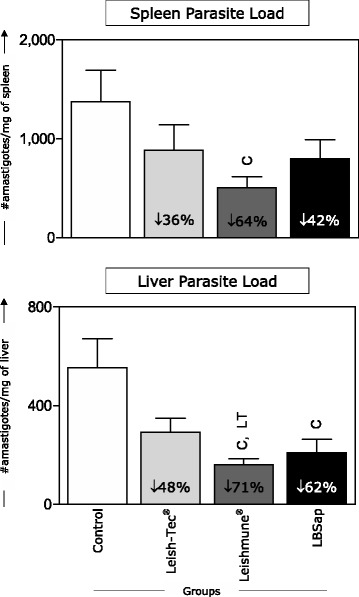
Comparative parasite burden in spleen and liver samples determined 30 days after experimental *L. infantum* challenge in BALB/c mice immunized with the multicomponent LBSap vaccine, Leish-Tec® or Leishmune®. The x-axis displays the different analyzed groups (*n* = 5 mice/group in two independent batches): Control (□); Leish-Tec® (); Leishmune® (); and LBSap (killed *L. braziliensis* vaccine plus saponin; ■). The y-axis represents the mean values (with standard deviations) of amastigote numbers per milligram of organ. The percentage of parasitism reduction in relation to the Control group is represented in each bar. Significant inter-group differences are marked by letters (C: Control group and LT: Leish-Tec® group)

As shown in Fig. 6 (upper panel), a 64 % reduction of amastigotes in the spleen was observed in the Leishmune® group (*P* = 0.034) when compared with the Control group. Moreover, regarding the spleen parasite burden, there was a decrease of 36 % in the Leish-Tec® group and 42 % in the LBSap group (Fig. 6, upper panel).

Regarding parasite burden in the liver (Fig. 6, bottom panel), there was a reduction of 71 % in the Leishmune® group (*P* = 0.004) and of 62 % in the LBSap group (*P* = 0.036) when compared with the Control group. Additionally, the Leish-Tec® group experienced a decrease of 42 % in parasite burden in the liver (Fig. 6, bottom panel). Interestingly, a parasite load reduction of 23 % in the Leishmune® group was observed as compared with the Leish-Tec® group (*P* = 0.044) (Fig. 6, bottom panel).

## Discussion

In the past years, several efforts have been made to improve the scientific knowledge regarding the immunological tools to control CVL, mainly focusing on the characterization and test of distinct *Leishmania*-derived antigens as vaccines and candidates for VL control. Regardless of these efforts, there are still relevant gaps about the immunological mechanism of protection triggered by distinct vaccines and novel immunobiological candidates for VL control. Such information would provide insights to improve the effectiveness of VL immunoprophylaxis [4, 21].

The murine experimental model for *Leishmania* infection represents a useful tool to investigate aspects related to parasite-host interactions and has significantly contributed to the design of hypothesis of immune-mediated mechanisms relevant to understand distinct aspects of leishmaniasis as well as identified relevant elements associated with protective response in immunoprophilactic approaches. However, although the data generated in murine models can be used to predict a hypothesis relevant to other host species, it is important to mention that discussion of mouse model-derived results must be performed carefully, taking proper prudence to avoid over interpretation. In this sense, the data generated in the present investigation must be further validated in complementary investigations performed in dogs, since these hosts present particularities in their immune response that are distinct from those observed in mouse models and critical for protection against *Leishmania* infection. Nevertheless, there are several findings previously reported in murine models that were further validated in dog hosts.

Efficient immunization against infectious agents requires the participation of the innate immune response [22]. In this study, we observed a neutrophil reduction at 15^AVac^ with respect to the BV in Leish-Tec® and LBSap groups and the re-establishments of population levels at 30^AChal^. The role of neutrophils in the *Leishmania* infection is still not fully understood. It has been described that neutrophils play a role in the establishment of infection as “Trojan horses” [23]. However, some authors believe that the neutrophils can also be associated with the parasite control during ongoing VL [24–26].

Immunophenotypic analysis showed an increase in monocyte (CD14^+^) frequency in the Leish-Tec® (30^AChal^) and Leishmune® groups (15^AVac^), despite the reduction found in the LBSap group (15^AVac^). Increased amounts of circulating CD14^+^ monocytes were associated with asymptomatic disease compatible with parasite control in VL [27] or high immunogenicity elicited by vaccination against CVL [16]. In fact, monocytes/macrophages are also extremely important in performing phagocytosis, killing pathogens through the respiratory burst, and participating in the production of proinflammatory cytokines [28]. All immunized groups displayed increase in NK cell frequency, particularly after experimental challenge (30^AChal^). It has been proposed that these cells have the ability to induce IL-12 production, favoring high levels of nitric oxide production, activation of macrophages, and thus inducing leishmanicidal activity and parasite control [29].

The innate and adaptive immune systems synergize to activate and execute a protective immune response following vaccination. However, the adaptive immune system allows the host to generate both antigen-specific responses and immunological memory, which have primary importance in the effective response against pathogens [30].

Regarding the CD19^+^ B-lymphocyte levels, a reduction in the LBSap group (15^AVac^ and 30^AChal^) was observed. It is possible to speculate that this reduction is related to the migration of these cells to lymphoid organs such as the spleen for polyclonal activation and antibody production, as previously reported [16, 31]. In agreement with this hypothesis, we observed high increases in IgG and IgG1 (15^AVac^ and 30^AChal^) in all immunized groups, indicating similar high antigenicity elicited by analyzed vaccines. Furthermore, the Leish-Tec® group displayed lower IgG2a production at 15^AVac^ compared to BV and compared to other vaccine groups. To date, there is no data in the literature using the Leish-Tec® commercial formulation in mouse models. It has been reported that IgG2a is closely related to the type I immune response and type 2 response correlates with IgE and IgG1 [32, 33]. It has been proposed that one advantage of Leish-Tec® is the fact that it induces the production of anti-A2, but not anti-SLA antibodies [10] that would allow the discrimination of immunized from infected dogs. However, it has been shown that Leish-Tec®-immunized dogs present IgG, IgG1 and IgG2 reactivity to SLA antigen. In fact, using a large number of dogs (*n* = 39) at different times after vaccination, Fernandes and colleagues have demonstrated that the IgG seroconversion rate of Leish-Tec®-immunized dogs can reach 59.5 %, as early as 21 days after the first dose and 54.8 % later on at 21 days after the second dose [34]. These findings corroborate the data observed in the present investigation showing the ability of Leish-Tec® vaccination to elicit the production of anti-SLA IgG.

Regarding the frequency of CD4^+^ T lymphocytes, we observed increased levels in the Leish-Tec® (30^AChal^), Leishmune® (30^AChal^) and LBSap (15^AVac^ and 30^AChal^) groups. Because the memory T cells are critical for inducing protection against infections in the long-term [35], high levels of these cells after the vaccine protocol and experimental challenge may be associated with immune cell-mediated protection. The CD4^+^ T-cell induction in anti-VL vaccines has been associated with high protection levels due to the ability of these lymphocytes to produce IFN-γ and promote macrophage activation favoring the leishmanicidal activity [35–37]. Additionally, it has been reported that dogs vaccinated with LBSap showed an increase in the frequency of circulating T cells and their subsets (CD4^+^ and CD8^+^) after the immunization protocol [16] and remains even after long-term experimental challenge in dogs [18]. The continued increase in the ratio of T-lymphocytes to B-lymphocytes (T/B ratio) in both Leish-Tec® and LBSap groups, after experimental challenge (30^AChal^), was associated with CD3^+^ T cells, particularly CD4^+^ T cells, with a consequent reduction in CD19^+^ B-cells. Moreover, we also observed an increased CD4/CD8 ratio at 30^AChal^ in the Leish-Tec® and LBSap groups that was related to the increased frequency of CD4^+^ cells. The increased CD4/CD8 T-cell ratio could favor parasite control, because CD4 T cells have been associated with an immune cell-mediated protective mechanism [4, 16, 18, 36].

The IL-2 increase in Leishmune® and LBSap and the increase in IL-6, TNF-α, and IFN-γ levels in LBSap may have contributed to the protection found in the vaccinated groups.

In fact, the analysis of overall proinflammatory and regulatory cytokine patterns, demonstrated by radar charts, indicated a prominent type 1 immune response (high producers of IL-6, TNF-α, IFN-γ, and IL-17) in the Leishmune® group and especially in the LBSap group. Additionally, Leish-Tec® presented a low frequency of IL-10 producers, which should contribute to the proinflammatory microenvironment. It is noteworthy that an effector immune response against VL, with the production of mainly proinflammatory cytokines such as IFN-γ and IL-12, is usually correlated with a type 1 response [38, 39]. In contrast, a predominantly type 2 immune response with regulatory cytokines, such as IL-10 and IL-4, appears to exacerbate the *Leishmania* infection [40, 41].

The parasitological data regarding the LBSap group corroborate with recent studies indicating that dogs immunized with this vaccine and challenged had a 54 % reduction in parasite load in splenic tissue [18]. The Leishmune® vaccine showed the highest parasite control in both spleen and liver. Previous studies demonstrated an 84.4 % reduction in liver parasite load in BALB/c mice immunized with FML plus saponin [42]. BALB/c mice immunized with A2 administered with *Propianibactrium acne*s as adjuvant and infected by *L. donovani* showed an 89 % reduction in liver parasites [43].

Together, our findings demonstrated evidences indicating the existence of particular phenotypic and functional features elicited by the three vaccines. However, all three vaccines are able to elicit relevant immunological changes supportive of putative anti-*Leishmania* protective mechanisms, such as a shift of cytokine balance towards higher IFN-γ/IL-10 ratio. In this context, the LbSap vaccine triggers an immune response comparable with other high performance vaccines already developed to control *L. chagasi* infection. Therefore, the LbSap vaccine represents a putative candidate to be further improved to meet the requirements of phase III clinical trials.

## Conclusions

The dataset led to the conclusion that the LBSap vaccine displayed immunological and parasitological profiles similar to other commercially available anti-CVL vaccines. In this sense, our data indicate that the LBSap vaccine presented the following: high antigenicity with sustained production of anti-*Leishmania* total IgG, IgG1, and IgG2a; prominent cellular immune response displaying increased levels of CD4^+^ T cells; and a proinflammatory cytokine profile with high levels of *Leishmania*-specific IL-6, TNF-α, and IFN-γ that contributed to a reduction in parasitism.

## Additional file

Additional file 1:
**Figure S1.** Representative flow cytometry pseudocolor plots illustrating the gate strategy applied to identify and select the circulating lymphocytes based on their morphometric profile (Size – forward scatter and granularity – side scatter) followed by the analysis of immunophenotypic features to quantify innate (CD3^-^CD49b^+^ NK-cells) and adaptive immunity cells (CD19^+^ B-cells; CD3^+^, CD3^+^CD4^+^ and CD3^+^CD8^+^ T-cells). A combination of morphometric (SSC) and immunophenotypic features (anti-CD14 staining) was applied to quantify CD14^+^ monocytes. (PDF 504 kb)

## Abbreviations

15^ASaline^: 15 days after the third saline
15^AVac^: 15 days after the third vaccination
30^AChal^: 30 days after experimental challenge
BV: Before the first vaccination
C: Control group
CVL: Canine visceral leishmaniasis
GAPDH: Glyceraldehyde 3-phosphate dehydrogenase; Abbreviated as horseradish peroxidase
HRP: horseradish peroxidase
LB: LBSap group
LBSap: *Leishmania braziliensis* antigen and saponin adjuvant
LM: Leishmune® group
LT: Leish-Tec® group; natural killer cell
PCR: Polymerase chain reaction
SLcA: Soluble lysate of *L. infantum* antigen
VL: Visceral leishmaniasis
