# Supplementary figures and images for: Multicomponent LBSap vaccine displays immunological and parasitological profiles similar to those of Leish-Tec® and Leishmune® vaccines against visceral leishmaniasis
Source: Parasit Vectors. 2016 Aug 30;9(1):472. doi: 10.1186/s13071-016-1752-6 (PMC5006379; doi:10.1186/s13071-016-1752-6)

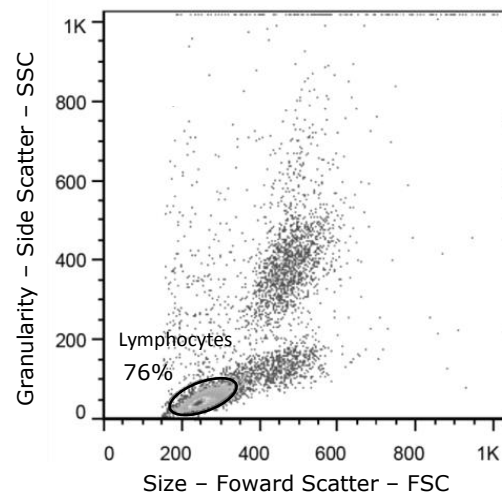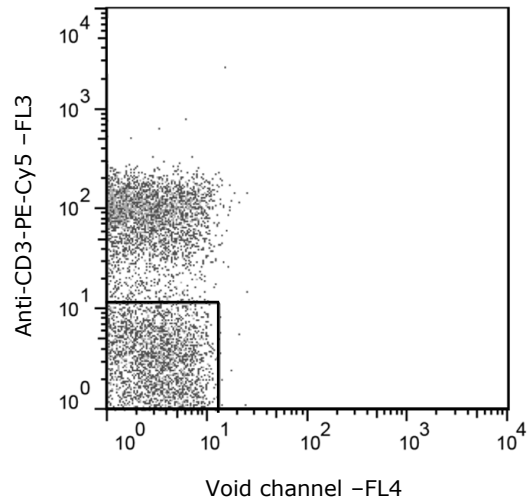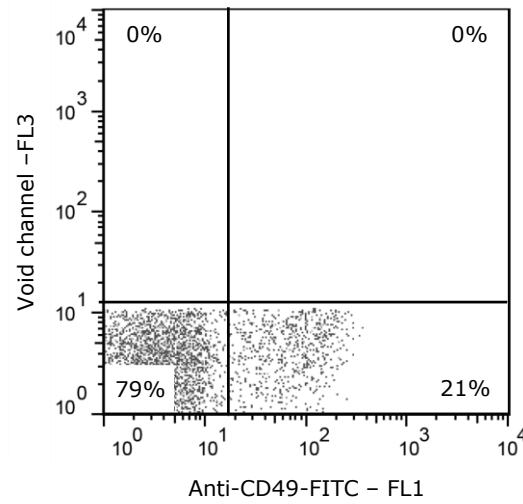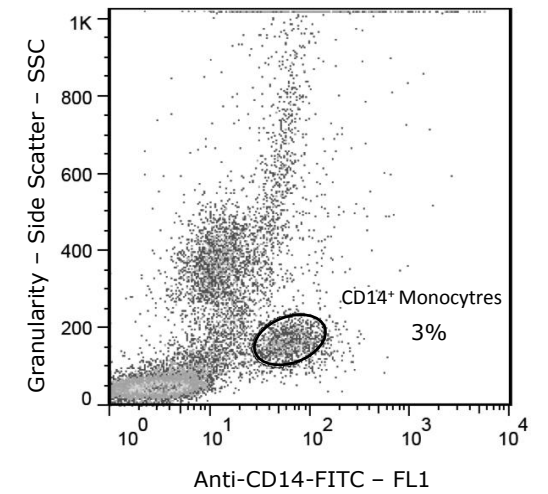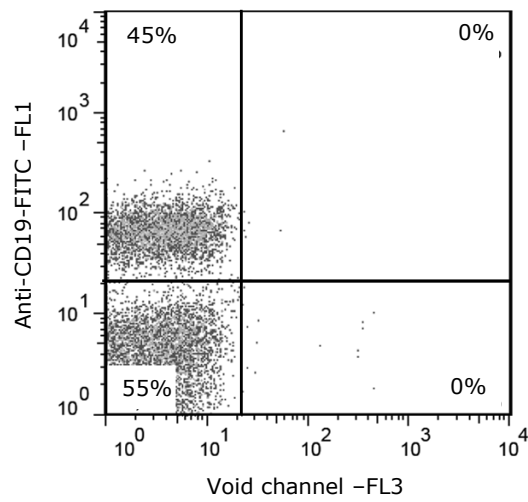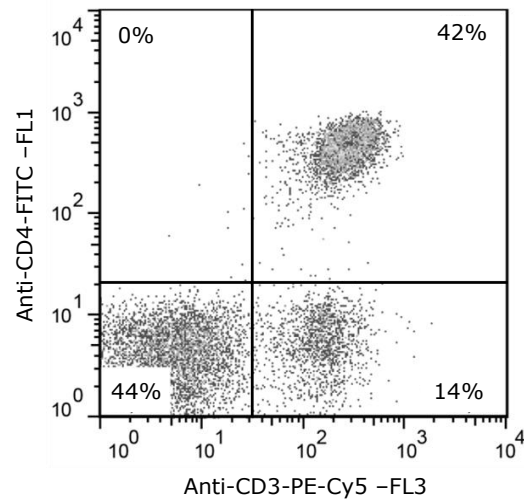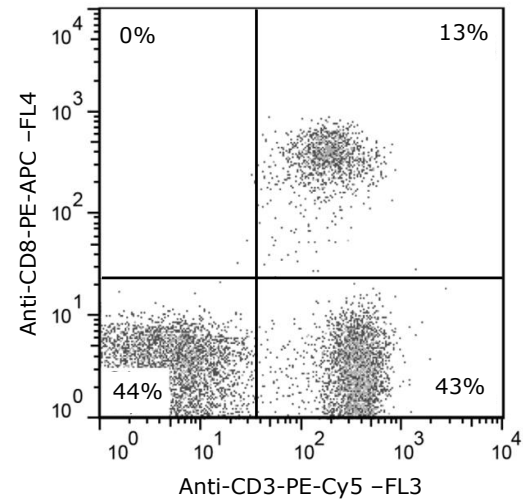

Supplement: Additional file 1: — Figure S1. Representative flow cytometry pseudocolor plots illustrating the gate strategy applied to identify and select the circulating lymphocytes based on their morphometric profile (Size – forward scatter and granularity – side scatter) followed by the analysis of immunophenotypic features to quantify innate (CD3-CD49b+ NK-cells) and adaptive immunity cells (CD19+ B-cells; CD3+, CD3+CD4+ and CD3+CD8+ T-cells). A combination of morphometric (SSC) and immunophenotypic features (anti-CD14 staining) was applied to quantify CD14+ monocytes. (PDF 504 kb) [file 13071_2016_1752_MOESM1_ESM.pdf]
